# Early childhood adversity and non-affective psychosis: a study of refugees and international adoptees in Sweden

**DOI:** 10.1017/S003329172100355X

**Published:** 2023-04

**Authors:** Anders Hjern, Jesús Palacios, Bo Vinnerljung

**Affiliations:** 1 Centre for Health Equity Studies (CHESS) and Clinical Epidemiology/Department of Medicine, Karolinska Institutet/Stockholm University, Stockholm S 171 77, Sweden; 2 Department of Developmental Psychology, University of Seville, Seville, Spain; 3 Department of Social Work, Stockholm University, Stockholm S 106 91, Sweden

**Keywords:** Adoption, refugees, migration, psychosis, early childhood, adversity

## Abstract

**Background:**

Previous Scandinavian studies have shown increased levels of psychiatric morbidity in young refugees and international adoptees with an origin outside Europe. This study investigated their risk of non-affective psychotic disorders (NAPD) and whether this risk is influenced by early childhood adversity, operationalised as age at adoption/residency, and/or gender.

**Methods:**

Register study in Swedish national cohorts born 1972–1990 including 21 615 non-European international adoptees, 42 732 non-European refugees that settled in Sweden at age 0–14 years and 1 610 233 Swedish born. The study population was followed from age 18 to year 2016 for hospitalisations with a discharge diagnosis of NAPD. Hazard ratios (HRs) were calculated in gender stratified Cox regression models, adjusted for household income at age 17.

**Results:**

The adjusted risks of NAPD were 2.33 [95% confidence interval (CI) 2.07–2.63] for the international adoptees and 1.92 (1.76–2.09) for the former child refugees, relative to the Swedish-born population. For the international adoptees there was a stepwise gradient for NAPD by age of adoption from adjusted HR 1.66 (1.29–2.03) when adopted during the first year of life to adjusted HR 4.56 (3.22–6.46) when adopted at ages 5–14 years, with a similar risk pattern in women and men. Age at residency did not influence the risk of NAPD in the refugees, but their male to female risk ratio was higher than in Swedish-born and the adoptees.

**Conclusion:**

The risk pattern in the international adoptees gives support to a link between early childhood adversity and NAPD. Male gender increased the risk of NAPD more among the refugees.

## Introduction

Early childhood adversity, particularly in interaction with stressful life events, has been shown to increase the risk for psychosis independent of pre-existing genetic liability (Sideli et al., 2020). Growing evidence also supports links between childhood trauma and psychosis (Stanton, Denietolis, Goodwin, & Dvir, 2020), as well as a dose-response relationship between adverse childhood experiences and psychiatric morbidity (Bentall, Wickham, Shevlin, & Varese, 2012). For the development of psychosis, the specific type of childhood adversity seems less important than the number of adversities, with strong effects when they are overwhelming and persistent (Trauelsen et al., 2015).

A previous Swedish register study revealed that hospital admissions with a psychiatric disorder were about three times as common among international adoptees in youth as in the general population (Hjern, Lindblad, & Vinnerljung, 2002). Non-affective psychotic disorders (NAPD) have been well studied in national adoptees in Scandinavia (Bohman & von Knorring, 1979; Wicks et al., 2010) but to our knowledge this is the first study to investigate preadoption risk factors for NAPD in international adoptees. Together with different forms of adversity in the birth family, institutionalisation is a common experience in international adoption that represents a significant preadoption adversity (Trauelsen et al., 2015). A meta-analysis of outcomes after institutional upbringing showed that the length of time in institutions was associated with increased risk of developmental sequelae (van Ijzendoorn et al., 2020). Age at adoption may be seen as an expression of the duration of exposure to adversity and previous research has documented that negative psychosocial outcomes have a positive correlation with age at adoption (Hjern, Palacios, & Vinnerljung, 2018; Sonuga-Barke et al., 2017). Gender has not previously been found to play a moderating effect for psychiatric morbidity among adoptees (Behle & Pinquart, 2016).

Research evidence indicates that migration is a risk factor for the development of schizophrenia and other non-affective psychoses, although the differences with the majority population decrease considerably when socio-economic indicators are considered (Hjern, Wicks, & Dalman, 2004). A Swedish national cohort study (Hollander et al., 2016) and a meta-analysis (Brandt et al., 2019) show that, among migrants, refugees are at particular risk of developing psychosis, with refugee children being particularly vulnerable with regards to mental health. Longitudinal studies of refugee children in Scandinavia (Montgomery, 2011) have shown a high level of stress-related symptoms during the first years in exile, assumed to be related to the accumulation of stressful experiences of war and persecution in the country of origin (Almqvist & Brandell-Forsberg, 1997; Angel, Hjern, & Ingleby, 2001), an often lengthy asylum process (Montgomery & Foldspang, 2005) and the adjustment to a new and often disadvantaged socioeconomic environment (Hjern & Angel, 2000). Veling, Hoek, Selten, and Susser (2011) identified preschool age at migration as a risk factor for psychotic disorders in immigrants in the Netherlands, suggesting a link between adversity associated with migration during early childhood and psychotic disorder in adulthood. A British study (Kirkbride et al., 2017) found an increased risk for psychotic disorders with immigration in early school age (5–12 years), while Swedish (Dykxhoorn et al., 2019) and Danish (Pedersen & Cantor-Graae, 2012) register studies of psychotic disorders did not find any clear age at immigration patterns for child migrants.

Several studies have indicated that among refugees the risk for psychosis in general, and for NAPD in particular, is higher for male migrants than for females (Castillejos, Martin-Perez, & Moreno-Kustner, 2018; Hollander et al., 2016). The risk for psychosis in migrants in Europe has also been found to be higher for migrants from Africa (Castillejos et al., 2018; Hollander et al., 2016; Manhica, Hollander, Almquist, Rostila, & Hjern, 2016; Murray et al., 2020; Selten, van der Ven, & Termorshuizen, 2020) than for migrants from other continents.

Thus, international adoptees and child refugees both represent groups exposed to considerably more adversity during childhood than the general population in a welfare society like Sweden (Seeman, 2020). International adoptees are typically placed in well-resourced families previously vetted as suitable. Adversities associated with their preadoption environment, such as parental abuse/neglect and institutionalisation end with the adoption, although the duration of exposure to these adversities varies depending on age at placement. Many studies that have linked early childhood adversity to NAPD lack control for potential confounders related to exposure to adversity later in life (Sideli et al., 2020). Our adoption design tries to overcome this limitation. Child refugees also enter Sweden at different ages, but for them exposure to socioeconomic risk factors (e.g. material strains, lack of prospects, social isolation) persists thereafter (Hjern et al., 2004).

During large parts of the 1970' and 1980', Sweden had the highest per capita reception of international adoptees (Kane, 1993) and one of the most humanitarian refugee policies in Europe. In this study, we exploited this window of opportunity to study the impact of childhood adversity in international adoptees and child refugees on NAPD in the comprehensive Swedish high-quality national registers (Rosen, 2002). According to the dose-response perspective, our hypothesis is that adoptees with more prolonged exposure to adversity (adoption at higher ages) will have an increased risk for NAPD compared with those with shorter exposure. For refugees, we expected that the adversity associated with becoming a refugee in early childhood would lead to a higher risk than for child refugees who migrated at a later age. With reference to the research cited above, we will also compare risk patterns associated with geographic origin and gender in international adoptees and refugees.

## Method

This study was based on information from the Swedish national registers, containing data for psychiatric epidemiology with high validity and low attrition rates (Miettunen, Suvisaari, Haukka, & Isohanni, 2011). These registers are based on the unique personal identity number assigned to all Swedish residents at birth (or time of immigration), and data from different registers can be linked by use of these identity numbers. The linkage for this study was made by Statistics Sweden, a national state-funded government agency that produces official statistics and prepares register data for researchers. Before the data was made accessible for this research project, it was anonymised by replacing the personal IDs with random numbers and re-categorising variables that may be used to identify individuals in the dataset, like date of birth and country of origin, into broad categories. Because of the anonymisation and the large study population, obtaining informed consent was not possible. However, Swedish legislation makes it possible to access anonymised data from national registers for research under certain conditions, one being the approval of an approved ethics committee. This study was approved by the regional ethics committee in Stockholm region (No. 2014/415-31/5).

### Study groups

The study population was comprised of individuals born 1972–1990 who, according to the Register of the Total Population (Ludvigsson et al., 2016) were alive and residents in Sweden on their 18th birthday, had at least one adoptive or birth parent registered in the Multi-Generation Register (Ekbom, 2011) and no register record of emigration from Sweden. They were followed from their 18th birthday until 2016, when they were 26–44 years. Birth or adoptive parents of these individuals were identified in the Multi-Generation Register.

Information about calendar year of residency/adoption and country/region of birth was retrieved from the Register of the Total Population. Based on this information we created three study groups. *Non-European international adoptees* (*N* = 21 615) fulfilled the criteria of being born outside of Europe, having at least one Swedish-born adoptive parent and no birth parent in the Multi-Generation Register, and a recorded age of adoption below 15. *Non-European refugees* (*N* = 42 732) were born outside of Europe and had settled in Sweden before their 15th birthday and had no adoptive parent nor any Swedish-born parent in the Multi-Generation Register. Being a refugee was defined as having received residency as a refugee or as a family relative to a refugee, according to the national immigration and emigration database (STATIV, by Swedish acronym) for immigrants who received residency from 1986 and onwards, when this information was available. During 1972–85 being a refugee was approximated by a country/region of birth (Latin America, Ethiopia, Lebanon, Syria, Iraq, Iran) that made it probable that they were refugees according to Swedish migration statistics. In all, refugees that received residency during 1972–1985 made up 18.9% of the refugee study population. Finally, the *Swedish-born* consisted of all Swedish-born with a Swedish-born mother (*N* = 1 610 233) in the same birth cohorts.

### Outcomes

The dichotomised outcome variable, NAPD, was defined as being hospitalised at least once with a discharge diagnosis of NAPD after age 18, the youngest age of treatment within adult psychiatry in Sweden, according to the National Patient Register (Ludvigsson et al., 2011) from January 1991 to December 2016. NAPD was defined as a main or complimentary ICD-10 diagnosis at discharge in the interval F20 to-F29 during 1997–2016 or an ICD-9 diagnosis of 295, 297 or 298C-X during 1991–1996. Schizophrenia (F20) has been validated in this register source and found to have acceptable validity (Dalman, Broms, Cullberg, & Allebeck, 2002).

### Income and educational covariates

Gender and annual disposable household income were retrieved from the Longitudinal Integration Database for Health Insurance and Labour Market Studies (Ludvigsson, Svedberg, Olen, Bruze, & Neovius, 2019) in the year of the 17th birthday of the study population. Disposable household income was calculated by Statistics Sweden with an algorithm that includes all taxable income in the household deducted by paid taxes divided by consumer units, and further divided into quintiles by annual birth cohort of the entire study population.

The final year of compulsory education in a mainstream educational setting was identified in the National School Register. If no such year was present in the register, it was assumed that the education had been completed in a compulsory school for pupils with learning disabilities, the *special education* organisation for children with severe intellectual disabilities within the Swedish compulsory school system.

### Statistical analysis

Age-adjusted hazard ratios (HRs) with 95% confidence intervals (CIs) were estimated using Cox proportional hazards models of time in the study, with NAPD as the outcome variable. The analysis was stratified by gender since significant gender differences in risk (*p* < 0.001) were found in the refugee study group. Time in the study was calculated from the starting date, which was January 1991 (or at age 18 years), until the first hospital admission recorded in the National Patient Register, or date of death recorded in the National Cause of Death Register, or December 2016, whichever came first. Statistical analysis was conducted using SPSS [IBM SPSS Statistics version 25.0 (SPSS, Inc., IBM Corp., Armonk, NY, USA)].

In the analyses of age at adoption/immigration, this age was operationalised into four categories representing developmental significance and based on the distribution of this variable in the study groups (see Table 1). In the non-European international adoptees, the categories were 0, 1, 2–4 and 5–14 years, and year of immigration in the refugee population: 0–4, 5–9 and 10–14 years. In the multivariate analysis the data was analysed in two consecutive Cox regression models. Model 1 was adjusted for year of birth in four categories and Model 2 added household disposable income in quintiles.

**Table 1.** Sociodemographic characteristics of the study population

	Adoptees		Refugees		Swedish-born	
	*N*	%	*N*	%	*N*	%
Gender						
Male	9353	43.3	22 687	53.1	830 662	51.6
Female	12 262	56.7	20 065	46.9	779 571	48.4
Year of birth						
1972–1976	6121	28.3	6043	14.1	444 495	27.6
1977–1981	6449	29.8	10 372	24.3	390 739	24.3
1982–1986	6086	28.2	14 397	33.7	397 060	24.7
1987–1990	2959	13.7	11 940	27.9	377 939	23.5
Region of origin						
East Asia	4490	20.8	238	0.6		
South Asia	7739	35.8	840	2.0		
Other Asia				0.0		
Afghanistan	0	0.0	1093	2.6		
Vietnam	92	0.4	1785	4.2		
Other	2274	10.5	2200	5.1		
Middle East						
Iran	365	1.7	8788	20.6		
Iraq	0	0.0	9255	21.6		
Lebanon	64	0.3	4449	10.4		
Syria	0	0.0	2485	5.8		
Latin America						
Chile	1838	8.5	4188	9.8		
Other	4135	19.1	1989	4.7		
Africa						
Eritrea	0	0.0	577	1.3		
Ethiopia	461	2.1	1044	2.4		
Somalia	0	0.0	2162	5.1		
Other	157	0.7	1659	3.9		
Age at adoption/immigration						
0 year	8712	40.3	325	0.8		
1 year	7849	36.3	1490	3.5		
2–4 years	3890	18.0	8598	20.1		
5–9 years	1118	5.2	16 155	37.8		
10–14 years	46	0.2	16 184	37.9		
Disposable income (quintiles)						
1 (Low)	1871	8.7	27 133	63.5	256 153	15.9
2	2775	12.8	9065	21.2	322 586	20.0
3	3947	18.3	3805	8.9	339 766	21.1
4	5381	24.9	1876	4.4	346 133	21.5
5 (High)	7641	35.4	847	2.0	345 405	21.5
In special education						
Yes	921	4.3	2624	6.1	34 000	2.1
Total	21 615	100	42 732	100	1 610 233	100

These analyses were supplemented with two sensitivity analyses. Firstly, the age at adoption/immigration categories were analysed separately in the international adoptees and the refugees in a Cox regression with the inclusion of the variables in Model 2 defined above, and further including adjustment for region of origin. Secondly, we made an analysis in Model 2 defined above, with the exclusion of children who completed their compulsory education in a special education setting.

## Results

Descriptive socio-demographic information about the three study groups is presented in Table 1. There were more women, 56.7%, than men in the adoptee group, while the opposite was true for refugees, 53.1% men. The levels of disposable income were highest in the adoptee group and lowest among the refugees. East and South Asia and Latin America were the most common origins of the international adoptees, while the refugees most often originated in the Middle East and Latin America. Among the international adoptees, 40% were adopted during their first year of life, and another 36% during their second year. Among the refugees, 75% received residency at the age 5–14 years. In the general population, 2% completed their compulsory education within the special education system, while this was the case for 4% of the international adoptees and 6% of the refugees.

Table 2 shows the incidence of NAPD by study groups and covariates, for men and women separately. The overall incidence was 7.2/1000 for men and 5.0/1000 for women. The incidence was highest for men and women with education within the special education system: 22.1/1000 for men and 20.0/1000 for women. The incidence decreased gradually by increased disposable income in the household at age 17.

**Table 2.** Incidence of at least one hospital discharge with a diagnosis of NAPD by covariates

	Men			Women		
	*N*	Cases	1/1000	*N*	Cases	1/1000
Study groups						
Int adoptees	9353	139	14.8	12 262	135	11.0
Refugees	22 687	435	19.1	20 065	148	7.4
Swedish-born	830 662	5678	6.8	779 571	2781	4.9
Year of birth						
1972–1976	234 677	2003	8.5	221 982	1541	6.9
1977–1981	209 998	1670	8.0	197 562	1059	5.4
1982–1986	215 731	1498	6.9	201 812	879	4.4
1987–1990	202 296	1081	5.4	190 542	585	3.1
Household income						
Low						
Quintile 1	142 055	1565	11.1	143 102	1061	7.4
Quintile 2	172 137	1409	8.2	162 289	880	5.4
Quintile 3	180 256	1180	6.6	167 262	751	4.5
Quintile 4	183 884	1036	5.6	169 506	704	4.2
High						
Quintile 5	183 273	1060	5.8	169 620	668	3.9
In special education						
Yes	21 169	467	22.1	16 376	328	20.0
Total	862 505	6252	7.2	811 898	4064	5.0

The Cox regression analysis demonstrated crude HRs of NAPD of 2.16 (95% CI 1.92–2.44) for international adoptees and 2.48 (2.28–2.70) for the refugees. Adjusting for disposable household income at age 17 increased the HR point estimate slightly for international adoptees to 2.33 (2.07–2.63) while it decreased for refugees to 1.92 (1.76–2.09). Male and female adoptees had similar risk estimates, while refugee men had a higher risk, adjusted HR 2.30 (2.08–2.55) compared with refugee women adjusted HR 1.29 (1.09–1.53), *p* < 0.001 in an interaction analysis (online Supplemental Table S1).

Table 3 demonstrates the risk estimates in the refugee and international adoptee study groups by age of adoption/immigration. For the international adoptees, there was a gradual increase by age at adoption from HR 1.66 (1.21–2.27) for men and HR 1.55 (1.12–2.15) for women adopted during their first year of life, to HR 4.69 (2.91–7.54) for men and HR 4.37 (2.64–7.27) for women adopted at 5–14 years of age. For refugees, the HRs were similar between age of migration categories for both men and women.

**Table 3.** Hazard ratios of NAPD by age at adoption/residency

	Men		Women	
	Model 1 RR (95% CI)	Model 2 RR (95% CI)	Model 1 RR (95% CI)	Model 2 RR (95% CI)
Adoptees by age at adoption				
0 years	1.53 (1.12–2.10)	1.66 (1.21–2.27)	1.42 (1.03–1.98)	1.55 (1.12–2.15)
1 year	2.03 (1.51–2.72)	2.18 (1.63–2.93)	1.91 (1.42–2.56)	2.05 (1.53–2.76)
2–4 years	2.85 (2.07–3.92)	3.03 (2.20–4.17)	3.92 (2.86–5.37)	4.11 (3.00–5.64)
5–14 years	4.50 (2.80–7.25)	4.69 (2.91–7.54)	4.30 (2.59–7.14)	4.37 (2.64–7.27)
Refugees by age of residency				
0.4 year	2.77 (2.27–3.41)	2.21 (1.80–2.73)	1.50 (1.06–2.12)	1.26 (0.89–1.78)
5–9 years	3.05 (2.62–3.56)	2.36 (2.02–2.77)	1.68 (1.29–2.17)	1.34 (1.03–1.74)
10–14 years	3.09 (2.67–3.60)	2.30 (1.97–2.68)	1.68 (1.30–1.18)	1.27 (0.98–1.65)
Swedish-born	1	1	1	1
Year of birth				
1972–1976		1		1
1977–1981		1.05 (0.98–1.12)		0.89 (0.83–0.97)
1982–1986		1.12 (1.05–1.21)		0.90 (0.83–0.99)
1987–1990		1.21 (1.12–1.31)		0.86 (0.78–0.96)
Disposable household income at age 17				
Low				
Quintile 1		1.73 (1.60–1.88)		1.85 (1.68–2.04)
Quintile 2		1.40 (1.29–1.51)		1.38 (1.25–1.53)
Quintile 3		1.14 (1.29–1.51)		1.15 (1.04–1.27)
Quintile 4		0.98 (0.90–1.07)		1.06 (0.96–1.18)
High				
Quintile 5		1		1

Table 4 presents an analysis of covariates stratified by study group. In the large Swedish-born study group, there was a clear gradient by quintiles of disposable household income with higher risk in the lower income quintiles. The refugees had the lowest female to male adjusted HR, with 0.42 (0.36–0.48), compared with adjusted HR 0.75 (0.59–0.96) for adoptees and adjusted HR 0.70 (0.67–0.73) for Swedish-born. African origin had the highest HRs in both adoptees and refugees, with HRs of 2.20 (1.31–3.71) and 2.47 (1.87–3.26), respectively, relative to an origin in Latin America.

**Table 4.** Incidence and hazard ratios of NAPD by age at adoption/residency, region of origin and covariates stratified by study group

	Adoptees		Refugees		Swedish-born	
	Incidence (%)	RR (95% CI)	Incidence (%)	RR (95% CI)	Incidence (%)	RR (95% CI)
Age at adoption						
0 year	0.8	1				
1 year	1.1	1.33 (0.98–1.82)				
2–4 years	1.9	2.24 (1.63–3.10)				
5–14 years	2.5	3.09 (2.02–4.72)				
Age at residency						
0–4 years			1.3	1		
5–9 years			1.2	1.01 (0.84–1.22)		
10–14 years			1.6	0.98 (0.81–1.19)		
Region of origin						
East Asia	0.9	0.76 (0.52–1.12)	0.1	0.43 (0.06–3.11)		
South Asia	1.2	1.05 (0.78–1.43)	1.4	1.47 (0.85–2.55)		
Other Asia	1.2	1.00 (0.64–1.55)	1.2	0.86 (0.60–1.22)		
Africa	3.3	2.20 (1.31–3.71)	3.3	2.47 (1.87–3.26)		
Middle East	2.4	1.84 (0.94–3.62)	1.2	0.94 (0.73–1.21)		
Latin America	1.2	1	1.4	1		
Gender						
Male	1.5	1	2.1	1	0.7	1
Female	1.1	0.75 (0.59–0.96)	0.8	0.42 (0.36–0.48)	0.5	0.70 (0.67–0.73)
Year of birth						
1972–1976	1.3	1	2.1	1	0.7	1
1977–1981	1.2	1.04 (0.76–1.43)	1.6	0.98 (0.76–1.26)	0.6	0.97 (0.93–1.09)
1982–1986	1.2	1.23 (0.88–1.77)	1.3	0.95 (0.74–1.22)	0.5	1.03 (1.06–1.09)
1987–1990	0.9	1.35 (0.87–2.10)	1.0	0.86 (0.64–1.15)	0.4	1.07 (1.00–1.14)
Disposable household income at age 17						
Low						
Quintile 1	1.1	0.90 (0.58–1.40)	1.1	1.28 (0.66–2.49)	0.7	1.85 (1.73–1.97)
Quintile 2	1.2	1.00 (0.69–1.47)	1.0	1.22 (0.62–2.41)	0.5	1.41 (1.32–1.50)
Quintile 3	1.0	0.80 (0.56–1.15)	1.2	1.36 (0.67–2.75)	0.4	1.15 (1.08–1.23)
Quintile 4	1.1	0.98 (0.72–1.33)	1.0	1.34 (0.63–2.86)	0.4	1.01 (0.95–1.09)
High						
Quintile 5	1.1	1	0.5	1	0.4	1

A sensitivity analysis was performed with the exclusion of children in the special education system, to investigate whether there were indications of selection of children with intellectual disabilities into the older age groups at adoption (see Table 5). The percentage of adoptees who completed their compulsory education in a special education setting increased gradually with age at adoption; from 2.5% at adoption age 0 years to 9.9% at adoption age 5–14 years. Accordingly, risk estimates relative to the Swedish comparison population decreased slightly in all ages among adoptees when children in special education were excluded, particularly among those adopted after age 5 years, but remained considerably higher for those adopted after age 2 years compared with those adopted at an earlier age.

**Table 5.** Hazard ratios of NAPD by age at adoption, with and without the inclusion of pupils in the special education organisation

	Special education	All	Excluding special education
	%	RR (95% CI)^a^	RR (95% CI)^a^
Adoptees by age at adoption			
0 year	2.8	1.61 (1.29–2.03)	1.47 (1.15–1.86)
1 year	4.3	2.14 (1.73–2.63)	1.95 (1.56–2.42)
2–4 years	5.7	3.52 (2.81–4.40)	3.27 (2.58–4.15)
5–14 years	9.9	4.56 (3.22–6.46)	3.15 (2.01–4.94)
Refugees	6.1	1.92 (1.76–2.09)	1.91 (1.74–2.09)
Swedish-born	2.1	1	1
Gender			
Male	2.5	1	1
Female	2.0	0.68 (0.65–0.71)	0.68 (0.65–0.71)

a Model adjusted for year of birth and disposable income.

## Discussion

In this register study of hospitalisation with a discharge diagnosis of NAPD in adult Swedish national cohorts, we found a twofold increased risk for two categories of migrants with an origin outside Europe, international adoptees and former child refugees. For the adoptees the risk increased gradually with age at adoption, while no influence of age at residency was found for the former child refugees. Gender had only a negligible influence on this risk in the adoptee group relative to the native comparison population, but in the refugee group there was a considerably greater male to female risk ratio relative to the Swedish-born. African origin was associated with a higher risk among both adoptees and refugees.

The graded association of age at adoption with NAPD gives support to our hypothesis regarding links between the burden of early adversity and NAPD. The sooner the adoptees had been lifted out of their preadoption living conditions, the lower was their risk, with risk increasing three to fourfold for those adopted after age 2 years compared with those adopted during their first year. Research has consistently documented the relation between the burden of adverse childhood experiences and enduring health, emotional and behavioural problems (Hughes et al., 2017; Paine, Fahey, Anthony, & Shelton, 2021; Petruccelli, Davis, & Berman, 2019). These adverse experiences are very often present during the time with birth parents (malnutrition, neglect, abuse) and while in institutional care, research indicating a strong positive association between the number of adversities and the age at adoptive placement (Anthony, Paine, & Shelton, 2019).

Meta-analytical evidence has shown that disorganised attachments and difficulties in forming secure attachments are more often present in adopted children than in the normative samples, when adoption happens after the first year of life (van den Dries, Juffer, van Ijzendoorn, & Bakermans-Kranenburg, 2009). In a systematic review, Sideli et al. (2020) reported evidence for considering insecure attachment as a mediator between childhood adversity and psychosis.

Global deficits in executive functioning have been suggested as another pathway between severe childhood adversity and psychopathology later in life (Wade, Zeanah, Fox, & Nelson, 2020). In the English and Romanian Adoption Study, Romanian adoptees with more prolonged institutional deprivation (over 6 months) had persistently higher rates than UK controls of inattention, hyperactivity and cognitive impairment from childhood to young adulthood (Sonuga-Barke et al., 2017). Relatedly, a Swedish register study has shown an increased risk for ADHD in international adoptees at ages of 6–21 years (Lindblad, Ringbäck Weitoft, & Hjern, 2010), with a more than twofold increased risk in children adopted after age 2 years compared with those adopted during their first year. Further, ADHD in school age has been shown to be associated with an increased risk for a psychotic disorder in adulthood (Nourredine et al., 2021).

Contrary to our hypothesis, age at migration did not influence the risk of NAPD among the former child refugees. This contradicts the results from Veling and Susser (2011), who found a gradual decrease of the risk for developing a psychotic disorder with increasing age of migration in their Dutch study of immigrant children and a British study that found an increased risk for early school age at migration (Kirkbride et al., 2017), but is in line with previous Scandinavian studies (Dykxhoorn et al., 2019; Pedersen & Cantor-Graae, 2012). We speculate that the diverging results might be explained by differences in the composition of the study populations, where the Scandinavian study groups consisted mostly of refugees while the Dutch and British study groups primarily consisted of labour migrants from former colonies and high-income countries. It is reasonable to believe that individuals in a child refugee group more often have been exposed to more serious and persistent adversities before leaving their country (armed conflict, persecution), during the flight and in the transition to the new country during the asylum inquiry than study populations where immigration is planned and voluntary. Thus, for forced migrants, the greater burden of adversity, that affects all age groups, may decrease the importance of young age at migration. This interpretation finds support from some previous research that has shown a higher risk of psychosis in refugees compared with other immigrants (Brandt et al., 2019; Hollander et al., 2016). As in previous Swedish research (Hjern et al., 2004; Hollander et al., 2016), the risk of NAPD in the refugees compared to the native population was attenuated considerably when the analysis was adjusted for disposable household income at age 17 years.

Another specific finding in the refugee group was the higher risk of NAPD in men compared to women relative to the adoptees and the Swedish born population. This pattern replicates the gender pattern found in a previous Swedish study of NAPD in a younger and more recent cohort of adult refugees (Hollander et al., 2016). The discrepancy in the gender pattern between the refugees and the adoptees in our study seems to indicate that this has more to do with the context in Sweden than with the specific stressful circumstances for each group in the country of origin. International adoptees are raised in a Swedish cultural context, while the refugees are raised in a family context which often is more like that of the country of origin when it comes to gender roles and expectations. We can only speculate about the specific contextual differences here, such as culturally based gender expectations of teenage risk behaviours associated with increased risk of NAPDs. One such risk behaviour could potentially be cannabis use during the teens. A meta-analysis by Kiburi, Molebatsi, Ntlantsana, and Lynskey (2021) has shown that such abuse increases the risk of a psychotic disorder by around 70%. In a Swedish study of health behaviours in teenagers, first generation non-European immigrant boys were found to use illicit drugs around twice as often as non-European immigrant girls, while boys and girls in the native population used drugs in a similar manner (Svensson & Hagquist, 2010). Further studies are needed to clarify these intriguing gender patterns.

The only geographic region that was associated with an increased risk for NAPD in both international adoptees and refugees in this study was Africa. The same finding has been reported for risk of schizophrenia in a similar Swedish register material (Manhica et al., 2016). Most refugees as well as international adoptees in Sweden with an African background originate in East Africa, thus opening up for shared health related risk factors like childhood perinatal complications, infections and malnutrition (Manhica et al., 2016). The experience of being victims of discrimination and racism in daily life is another possible shared risk factor (Veling & Susser, 2011) between adoptees and refugees with an origin in Africa. Since the 1980' it has been debated in the UK whether there is also discrimination within the psychiatric services so that skin colour influences the risk of being misdiagnosed with a psychotic disorder (Sharpley, Hutchinson, McKenzie, & Murray, 2001). Lately, this possibility has also been discussed in the US, where experimental evidence suggests that being Afro-American increases the possibility of being diagnosed with a psychotic disorder compared with other patients with a similar symptomatology (Londono Tobon et al., 2021). No such studies have yet been performed in the Nordic countries.

### Strengths and limitations

The study exploited the unique possibilities in the high quality Swedish national registers to create the large national cohorts of international adoptees and child refugees necessary to study a low frequency outcome like NAPDs. Considering the timing of adversity, our adoption design has the advantage of a distinct focus on early childhood experiences. Most important, our approach limits the problem of confounding due to exposure to adversity later in life, which is a major problem in many studies on this topic (Sideli et al., 2020). The adoptive families on average have a better socioeconomic situation then the general population, and before adoption have been vetted by child welfare professionals regarding major psychological, psychiatric and social problems that may interfere substantially with parenting capacities.

The information about age at adoption available to the study was based on whole calendar years. Hence, the precision of the categories used in the study was quite low with considerable overlaps, particularly for the two youngest age groups. Thus, it seems likely that the study underestimated the true risk differences between the age at adoption categories. For the refugees, the age at migration categories were even less precise, since the age at residency was based on the year when residency was granted and not when they set foot on Swedish soil.

The lack of information about the preadoption circumstances of the international adoptees opens the possibility that the association between age at adoption and NAPD could be created by a selection of children with disabilities into the older age at adoption groups. To investigate that possibility, we made a sensitivity analysis where we excluded children who had had their compulsory education in a special education setting. As expected from research on children exposed to institutional deprivation (Sonuga-Barke et al., 2017), the percentage of children in special education settings increased with age at adoption, but even for those adopted after age 5, it was lower than 10%. When we excluded those in special education, the risk estimates decreased somewhat in all age groups, and more in those adopted after 5 years of age, but the general pattern of a clear hierarchy of age at adoption remained. Thus, we do not believe that such selection was likely to explain our main results.

## Conclusions

The risk of NAPD increased gradually with age at adoption thereby supporting the hypothesis that cumulative adversity during the first years of life is associated with the risk of NAPD in adulthood. For the refugee group, there was no association with age at migration, but indications of an increased risk associated with persistent socioeconomic disadvantage. The considerably higher male to female risk ratio for NAPD in the refugees compared with the other study groups is intriguing and calls for further research. Regardless of causality issues, international adoptees that were adopted late in their childhood, and male refugee youth, should be viewed as risk groups for NAPD, particularly if they have been exposed to severe and persistent adversities.

## Supplementary material

For supplementary material accompanying this paper visit https://doi.org/10.1017/S003329172100355X. click here to view supplementary material
