# Supplementary material for: Early childhood adversity and non-affective psychosis: a study of refugees and international adoptees in Sweden
Source: Psychol Med. 2021 Sep 2;53(5):1914–23. doi: 10.1017/S003329172100355X (PMC10106297; doi:10.1017/S003329172100355X)
Supplement: Supplementary file 1 [file S003329172100355Xsup001.docx]

Supplementary Table S1. Hazard ratios of NAPD by study group.

|  | All | | Men | | Women | |
| --- | --- | --- | --- | --- | --- | --- |
|  | Model 1^1^  RR (95% CI) | Model 2^2^  RR (95% CI) | Model 1  RR (95% CI) | Model 2^2^  RR (95% CI) | Model 1  RR (95% CI) | Model 2^2^  RR (95% CI) |
| International adoptees | 2.16 (1.92-2.44) | 2.33 (2.07-2.63) | 2.15 (1.81-2.54) | 2.31 (1.95-2.573 | 2.17 (1.83-2.57) | 2.33 (1.96-2.76) |
| Refugees | 2.48 (2.28-2.70) | 1.92 (1.76-2.09) | 3.01 (2.73-3.32) | 2.30 (2.08-2.55) | 1.64 (1.39-1.93) | 1.29 (1.09-1.53) |
| Swedish-born | 1 | 1 | 1 | 1 | 1 | 1 |

^1^Model 1 is adjusted for gender

^2^Model 2 is adjusted for gender. year of birth in four categories and disposable household income in quintiles
